# The Intersection of Giftedness, Disability, and Cultural Identity: A Case Study of a Young Asian American Boy

**DOI:** 10.3390/bs15081036

**Published:** 2025-07-30

**Authors:** Tammy Jean Byrd, Ty’Bresha Ebony Glass, Ophélie Allyssa Desmet, F. Richard Olenchak

**Affiliations:** 1 Department of Educational Studies, Purdue University, 610 Purdue Mall, West Lafayette, IN 47907, USA; glass29@purdue.edu (T.E.G.); olenchak@purdue.edu (F.R.O.); 2 Department of Educational Psychology, Ball State University, 2000 W University Ave, Muncie, IN 47306, USA; ophelie.desmet@bsu.edu

**Keywords:** multi-exceptionality, twice-exceptionality, giftedness, autism spectrum disorder, ADHD, cultural identity, talent development, case study

## Abstract

The present research examines the intersections of giftedness, disability status, and cultural identity through the case of Kent, a nine-year-old Asian American boy who is not only profoundly gifted but has also been diagnosed with autism spectrum disorder (ASD), attention deficit hyperactivity disorder (ADHD), and possibly developmental coordination disorder (DCD). This study offers a comprehensive exploration of how these overlapping factors shape Kent’s early talent development and educational experiences, while also highlighting the challenges faced by his family and their need for a personalized, holistic support system tailored to his unique combination of abilities and disabilities. While Kent’s case is not generalizable, it underscores the critical importance of understanding the dynamic interplay among giftedness, disability status, and cultural identity in developing effective educational strategies. Furthermore, we advocate for personalized interventions that extend beyond conventional approaches, such as applied behavior analysis (ABA), to adequately address the complex needs of multi-exceptional individuals like Kent.

## 1. Introduction

Existing research on twice- or multi-exceptionality has primarily focused on exploring the comorbidity of giftedness (or high ability) and a disability, with the aim of identifying strategies to improve educational experiences for students. While multi-exceptionality is acknowledged as a complex phenomenon, little research explores the intersection of giftedness, disability, and cultural identity in the early stages of talent development. This gap in literature is particularly pressing considering the growing cultural and ethnic heterogeneity within educational settings and the urgent need for culturally responsive approaches that effectively identify and nurture exceptional students. To address this critical gap in the literature, this manuscript presents a comprehensive, single-case study of a multi-exceptional, nine-year-old Asian American boy and his family. The child is not only gifted but also diagnosed with autism spectrum disorder (ASD) and attention deficit hyperactivity disorder (ADHD), illustrating the complexity of multi-exceptionality. This study is guided by the following research question:

How do giftedness, disability, and cultural identity collectively influence the early talent development and educational experiences of a multi-exceptional Asian American student?

This single-case study serves several important objectives that extend beyond individual case analysis to benefit broader social and professional practices. First, it aims to advance theoretical understanding of how giftedness, disability, and cultural identity intersect in early talent development, contributing to more nuanced models of multi-exceptionality that account for cultural diversity. Second, it seeks to identify systemic barriers to effective identification and support of culturally diverse, multi-exceptional students, revealing gaps in current educational and therapeutic approaches. Third, it aims to inform professional practice by demonstrating how multi-framework theoretical analysis can guide more comprehensive, culturally responsive assessment and intervention strategies. Finally, it contributes to educational equity by highlighting the unique challenges faced by multi-exceptional students from immigrant families and the need for culturally competent support systems. These objectives position this case study as a foundation for understanding complex intersectionality in multi-exceptionality, with implications for educator training, policy development, and intervention design for diverse learner populations.

### Theoretical Frameworks

This single-case study explores the talent development and educational experiences of Kent, a multi-exceptional Asian American student, through the lens of the following theoretical frameworks:

Bronfenbrenner’s (1979) ecological systems theory offers a comprehensive perspective on the interconnected environmental systems (i.e., microsystem, mesosystem, exosystem, macrosystem, and chronosystem) that shape Kent’s development. The microsystem includes Kent’s immediate surroundings, such as family, school, and peers. The mesosystem examines the interactions among these microsystems, while the exosystem encompasses external factors that indirectly influence his experiences, such as parental workplace policies or school board decisions. The macrosystem represents the broader cultural values, societal norms, and ideologies that shape his experiences as an Asian American student. Finally, the chronosystem accounts for the role of time and historical contexts in influencing his developmental trajectory.

Bronfenbrenner’s theory has been used to analyze how factors such as family structure, cultural identity, socioeconomic status, and school relationships act as barriers or supports for minority and at-risk gifted students. For example, a case study of a young, minority gifted learner utilized the ecological systems framework to examine how strong home–school connections and culturally sensitive educators facilitated the student’s ability to overcome obstacles and succeed in gifted programs (Stevenson, 2007). Bronfenbrenner’s bioecological model has also been used to explore identification issues and obstacles faced by underrepresented minority students in gifted education. The model helps researchers and educators understand how systemic and contextual influences affect access to gifted programs and the development of giftedness among minority populations (Crawford et al., 2019).

The interconnected systems in Bronfenbrenner’s model (1979) work in tandem with Gagné’s (2010) differentiated model of giftedness and talent (DMGT). This model distinguishes between giftedness (innate potential) and talent (developed competence), emphasizing the role of intrapersonal and interpersonal catalysts in nurturing natural abilities. By applying this framework, this study examines how Kent’s giftedness in specific domains interacts with his diagnoses of ASD and ADHD across environments, identifying factors that either facilitate or impede his talent development.

Furthermore, Kent’s identity as an Asian American student was explored through the lens of cultural ecological theory (Ogbu & Simons, 1998), which explains that the academic experiences of minoritized students are shaped by their interactions with the educational system and their responses to these interactions. Through this perspective, we aim to examine the influences on his identities—particularly his racial identity, given the “model minority” myth—on his educational journey and talent development. Additionally, this framework facilitates an examination of potential conflicts between cultural expectations and individual needs, and their impact on his socioemotional well-being.

Finally, due to negative teacher perceptions, the disharmony hypothesis (Neihart, 1999) was used to explore this case study. While rejecting the stereotype of the “mad genius”, this study acknowledges the socioemotional challenges that gifted students, especially those with disabilities, may encounter. This framework facilitates an exploration of how negative teacher perceptions, insufficient support, and the complexities of multi-exceptionality contribute to Kent’s internal conflicts and maladaptive behaviors. By highlighting the necessity of individualized socioemotional support, this framework emphasizes the need to address his unique challenges and foster positive developmental outcomes.

By integrating these theoretical perspectives, this study seeks to provide a comprehensive understanding of the dynamic interplay between giftedness, disability, and cultural identity in shaping the early talent development and educational experiences of a multi-exceptional Asian American student. This single-case study examines the strengths and challenges faced by Kent and his family as they navigate the complexities of multi-exceptionality within the context of their cultural background and the educational system. By exploring these interconnected factors through the lenses of Bronfenbrenner’s ecological systems theory, Gagné’s DMGT, cultural ecological theory, and the disharmony hypothesis, we aim to enhance the understanding of multi-exceptionality and inform strategies that better support this distinct group of diverse learners. By exploring these interconnected factors, we aim to enhance the understanding of multi-exceptionality and inform strategies that better support this distinct group of diverse learners.

## 2. Background

### 2.1. Multi-Exceptionality and Identification

Multi-exceptionality refers to individuals who possess exceptional abilities or strengths in areas such as intellectual, creative, artistic, or leadership skills, alongside one or more learning disabilities, disorders, or challenges (Foley-Nicpon et al., 2011; Reis et al., 2014/2014). This term has evolved from the earlier term “twice-exceptional”, reflecting a refined understanding that individuals can exhibit multiple areas of giftedness and challenge. These exceptionalities significantly impact students’ educational experiences. Multi-exceptional individuals may include gifted students with ADHD, highly creative individuals with dyslexia, or intellectually advanced individuals with ASD (Foley-Nicpon et al., 2013). The interplay of abilities and challenges in these individuals necessitates a comprehensive and individualized approach to identification and support, particularly when considering their cultural identities.

Recent scholarships have expanded the understanding of multi-exceptionality beyond the traditional twice-exceptional framework. Collins’s work on thrice-exceptional students provides valuable insights into the complexity of students who navigate multiple intersecting identities and exceptionalities (Collins, 2020). Similarly, Haines et al.’s exploration of the interaction between ability, disability, and cultural giftedness offers a foundational understanding of how these dimensions intersect to shape educational experiences (Haines et al., 2022). Our study builds upon this emerging literature by providing an in-depth case study that illustrates these complex interactions in practice.

Identifying multi-exceptional students can be complex. As DMGT suggests, the interaction between natural abilities (giftedness) and disabilities can mask a student’s true potential (Gagné, 2010). Traditional educational approaches may not fully address both aspects of their profiles, making it difficult to meet their educational needs. For instance, a child’s high mathematical ability might be overlooked due to his social communication challenges related to ASD. It is essential for educators and parents to recognize and support both aspects of a multi-exceptional individual, providing appropriate accommodations to help them reach their full potential (Amran & Majid, 2019). To achieve this, educators and parents must implement personalized educational strategies that account for both strengths and challenges and address social and emotional needs (Foley-Nicpon et al., 2013).

### 2.2. Autism Spectrum Disorder and Giftedness

Autism spectrum disorder (ASD) is a neurodevelopmental condition characterized by challenges in social interaction, communication, and restricted or repetitive behaviors (Desmet et al., 2024). Additionally, individuals with ASD may experience sensory sensitivities and difficulties adapting to change or maintaining routines. As a spectrum disorder, ASD can present a wide range of severity levels, characteristics, and associated strengths (Burger-Veltmeijer & Minnaert, 2011), creating a unique combination of strengths and challenges exhibited by multi-exceptional learners. These strengths and challenges go beyond academic achievement; gifted individuals with ASD may display intense focus, heightened attention to detail, or extraordinary memory skills, while simultaneously struggling with social interactions and communication.

While traits of giftedness—such as advanced cognitive abilities, rapid learning, and a deep passion for specific subjects—and ASD may appear distinct, their overlapping characteristics and behaviors frequently co-exist (Desmet et al., 2024). This can be seen through preferences for routine, a strong need for logical thinking, and an inclination toward solitary activities. However, the expression of these traits can differ among individuals, emphasizing the importance of recognizing individuality within this population (Desmet et al., 2024). Gifted students with ASD often exhibit complex profiles that require careful understanding and support from educators, parents, and professionals. Their talents and challenges may interact in intricate ways, necessitating tailored educational strategies and interventions (Desmet et al., 2024).

### 2.3. Attention Deficit Hyperactivity Disorder and Giftedness

Similarly, attention deficit hyperactivity disorder (ADHD) is a spectrum disorder characterized by persistent patterns of inattention, hyperactivity, and impulsivity that interfere with daily functioning and development (Lee & Olenchak, 2015). Individuals with ADHD often struggle with maintaining focus, organizing tasks, and regulating behavior, which can significantly impact various aspects of their lives, such as following instructions, completing assignments, and peer relationships (Gilman et al., 2013; Lee & Olenchak, 2015). Moreover, struggles with impulsivity and hyperactivity can manifest as restlessness and difficulty staying seated, impacting academic performance and social interactions (Foley-Nicpon et al., 2013). Nevertheless, as a spectrum disorder, some individuals may experience milder symptoms with minimal interference, while others may face pronounced challenges that significantly affect their academic, personal, and professional lives. 

The intersection of giftedness and ADHD creates an even more unique combination of exceptional abilities and challenges related to attention and self-regulation. While multi-exceptional individuals with ADHD may demonstrate heightened creativity and divergent thinking, the expression of overlapping characteristics such as high energy levels and rapid thinking may differ among individuals, emphasizing the need for personalized understanding and support (Baum & Owen, 2004). These intricate interactions of strengths and challenges necessitate tailored educational strategies and support that accommodate unique learning styles and address attention and self-regulation difficulties.

### 2.4. Cultural Implications

Meeting the needs of multi-exceptional individuals does not happen in a vacuum. It requires a personalized and holistic approach that not only considers individual characteristics, but also cultural and environmental contexts. Parents and caregivers play significant roles in talent development (Olszewski-Kubilius, 2002; Moon, 2003; Moon et al., 1998), especially when examining the cultural intersections in parenting styles. For example, in traditional Chinese culture, parental involvement in education is deeply rooted in Confucian values. Concepts such as “chiao shun” (training children in appropriate behaviors) and “guan” (to govern) highlight the significant influence of the home environment on a student’s educational experience (Yao et al., 2024; Chi, 2003; Fan & Chen, 2001; Chan, 2005).

This is particularly relevant for multi-exceptional Asian American students like Kent, where cultural expectations for academic achievement may interact with the challenges posed by ASD and ADHD. For instance, the emphasis on “chiao shun” might lead to increased pressure on Kent to conform to classroom expectations, even if those expectations are not aligned with his individual learning needs due to his disabilities. Failing to understand that certain cultural values may not be appropriate within certain contexts can be a source of socioemotional disharmony. Thus, misunderstanding and inadequate accommodations of cultural values can negatively impact educational and social outcomes for multi-exceptional learners, highlighting the importance of culturally responsive educational interventions that consider the interplay between cultural values, exceptional abilities, and disabilities.

#### 2.4.1. Immigration Status

The cultural implications of immigration status add further depth to the experiences of multi-exceptional students. According to Ogbu and Simons’s (1998) cultural ecological theory, minoritized students’ varying school performance stems from their interaction with the educational environment and their response to it. In this theory, Asian American students are often classified as voluntary immigrant minorities who approach school success with more optimistic expectations for the future; however, this only works to perpetuate the model minority myth and overlooks the unique needs of multi-exceptional students (Cooc, 2019; Park, 2019; Kiang et al., 2017; Park & Foley-Nicpon, 2022), such as the student in this case study. These implicit biases affect educator and parent perceptions of Asian and Asian American students, such as mistakenly attributing students’ challenges to laziness rather than underlying issues (Park et al., 2018), making it imperative to implement holistic approaches to talent development for multi-exceptional students. 

#### 2.4.2. Gender Identities

Gender identities also contribute to the complexity of cultural implications, varying widely across countries, historical contexts, religious doctrines, and contemporary societal changes (Chiu et al., 2015). In this study, we focus on Asian and Asian American cultures, where males have historically held the role of heads of households, serving as primary decision-makers and authority figures who ensure the stability and safety of the family (Xie, 2013). This patriarchal structure, in line with Ogbu and Simons’s (1998) theory, is often reinforced through an emphasis on education and strong encouragement to excel academically (Chiu et al., 2015). In this context, educational achievement is not only viewed as a personal milestone but also as a means of fulfilling familiar expectations and maintaining cultural values. 

Asian and Asian American perspectives may also differ due to various factors, such as immigration status and the influence of external cultures (Boer & Fischer, 2013). Traditional Asian social norms may dictate that males embody traits such as masculinity, strength, honor, respect, stoicism, and emotional restraint (Xie, 2013), with a fundamental emphasis on respecting and honoring family and elders (Chiu et al., 2015). Conversely, Asian Americans may adopt diverse perspectives of gender roles and identities, such as advocacy for balanced domestic and professional responsibilities (Yoshikawa et al., 2016). However, these evolving views are influenced by generational shifts, educational and socioeconomic factors, and individualized immigration experiences (Luo et al., 2013).

These factors, nested within cultural implications, become even more multifaceted when serving multi-exceptional students (Luo et al., 2013), who may experience comorbid disabilities that inhibit social interactions. These complexities further underscore the critical need for holistic and personalized approaches to education and talent development needed for multi-exceptional students to excel academically and effectively (Chiu et al., 2015).

### 2.5. Giftedness, Disharmony Hypothesis, and Dark Triad Traits

This study incorporates the disharmony hypothesis, which suggests that high cognitive ability may be linked to socioemotional adjustment difficulties among gifted and talented students (Gallagher, 1990; Neihart, 1999). While this concept has historically been associated with the myth of the “mad genius” (Lombroso, 1891), contemporary research indicates that giftedness itself does not inherently predispose students to socioemotional challenges. Rather, a variety of contextual factors contribute to these difficulties (Vaivre-Douret, 2011; Bailey, 2011; Peterson, 2009), including teacher attitudes (Jussim & Harber, 2005; Baudson & Preckel, 2013; Preckel et al., 2015).

Peterson (2011) asserted that teachers’ attitudes—particularly positive attitudes—can contribute to positive student outcomes. However, McCoach and Siegle (2007) found that teachers held a variety of attitudes towards gifted students and gifted education, with special education teachers often expressing less favorable views. This suggests that if positive attitudes contribute to better student outcomes, negative attitudes may lead to internal conflicts for gifted students, potentially resulting in adverse outcomes as well (Cross et al., 2016; Elhoweris, 2009).

For multi-exceptional students like Kent, these challenges can be further exacerbated. Negative teacher attitudes, combined with the complex needs associated with multipotentiality, can lead to inadequate support (Elhoweris et al., 2021; Geake & Gross, 2008). This lack of appropriate support may prompt maladaptive behaviors (Agaliotis & Kalyva, 2019; Eren et al., 2018; Neihart & Yeo, 2018) as students struggle to navigate their unique cognitive and socioemotional landscape.

By incorporating this perspective, the study aims to examine how negative teacher attitudes, inadequate support, and the challenges associated with multi-exceptionality may contribute to internal conflicts and potentially maladaptive behaviors in Kent. This framework underscores the importance of individualized socioemotional support to address the unique challenges of multi-exceptional students and promote positive outcomes.

### 2.6. Cultural Identity Development in Multi-Exceptional Contexts

Cultural identity, defined as an individual’s sense of belonging to and identification with their cultural group (Park et al., 2018), represents a critical yet understudied factor in the development of individuals with multiple exceptionalities. For Asian American students, cultural identity development involves navigating between heritage culture values and mainstream American cultural expectations, a process that becomes particularly complex when intersecting with exceptionalities.

Ethnic–racial identity development theory (Umaña-Taylor et al., 2004) proposes four identity statuses: diffuse (characterized by little exploration or commitment), foreclosed (characterized by commitment without exploration), moratorium (characterized by active exploration without commitment), and achieved (characterized by exploration leading to commitment). For multi-exceptional Asian American students like Kent, this developmental process may be complicated by how disabilities and giftedness are understood within different cultural frameworks.

Given Kent’s young age and his unique needs, we examined cultural identity through family narratives, cultural value conflicts, and Kent’s own expressions of cultural preference and belonging rather than any formal measures.

Kent’s case illustrates how cultural identity development in multi-exceptional students may follow non-linear pathways, where disability-related challenges create tensions with cultural expectations while giftedness may align with cultural values around academic achievement. Understanding these dynamics is crucial for providing culturally responsive support to students with multiple exceptionalities and their families.

## 3. Method

This study employs a qualitative, single-case study design, adhering to the methodological standards outlined by Yin (2014). A single-case study approach was selected to provide an in-depth and holistic understanding of the complex interplay between giftedness, disability, and cultural identity in shaping the early talent development and educational experiences of one multi-exceptional Asian American student. This approach allows for a rich, contextualized exploration of the research question: “How do giftedness, disability, and cultural identity collectively influence the early talent development and educational experiences of a multi-exceptional Asian American student?” This design is particularly well-suited for examining the complexities of Kent’s experiences through the lens of the chosen theoretical frameworks.

### 3.1. Theoretical Framework and Its Influence on Data Collection

The study is guided by Bronfenbrenner’s Ecological Systems Theory, Gagné’s differentiated model of giftedness and talent (DMGT), cultural ecological theory (Ogbu & Simons, 1998), and the disharmony hypothesis. These frameworks informed the data collection process in various ways. 

#### 3.1.1. Ecological Systems Theory

Bronfenbrenner’s (1979) ecological systems theory guided the exploration of multiple environmental levels influencing Kent. Interview questions were designed to elicit information about Kent’s interactions with family, teachers, and peers (microsystem); the collaboration between home and school (mesosystem); the impact of school policies (exosystem); and the broader cultural values and beliefs about giftedness and disability (macrosystem). For example, questions to parents explored their involvement in school activities, while interviews with school staff addressed school-wide policies affecting multi-exceptional students.

While Bronfenbrenner’s ecological systems theory provides a comprehensive framework for understanding environmental influences, it has faced several critiques. Neal and Neal (2013) argue that the theory’s emphasis on nested systems may oversimplify the complex, networked nature of contemporary social relationships. Additionally, some scholars critique the theory’s limited attention to individual agency and its potential for environmental determinism (Rosa & Tudge, 2013). Despite these limitations, the framework remains valuable for this study because it explicitly accounts for cultural macrosystems and temporal changes (chronosystem), which are essential for understanding the evolving experiences of immigrant families navigating educational systems.

#### 3.1.2. Differentiated Model of Giftedness and Talent

The use of the differentiated model of giftedness and talent (DMGT) shaped the investigation of Kent’s natural abilities (giftedness) and how these abilities are nurtured or hindered by intrapersonal factors (e.g., motivation, self-regulation) and environmental factors (e.g., educational opportunities, support systems) to develop talents. Diagnostic materials and school records were analyzed to identify Kent’s strengths, while interviews explored the support systems in place to foster his talents.

The DMGT, while influential, is not without controversy in the gifted education field. Critics argue that the model’s separation of giftedness and talent may create artificial distinctions that do not reflect the fluid, interconnected nature of ability development (Subotnik et al., 2011). Additionally, some scholars question whether the model adequately addresses the role of motivation and whether its focus on measurable outcomes may inadvertently privilege certain types of abilities (McClain & Pfeiffer, 2012). However, for multi-exceptional students, the model’s explicit attention to both facilitating and hindering catalysts provides a framework for understanding how disabilities and cultural factors can simultaneously support and impede development.

#### 3.1.3. Cultural Ecological Theory

The cultural ecological theory facilitated the examination of how Kent’s experiences as an Asian American student, who may be subject to stereotypes such as the model minority myth, shape his educational journey and talent development. Interview questions were designed to explore potential conflicts between cultural expectations and individual needs, and how these conflicts impact his socioemotional well-being. This included questions about family values, cultural identity, and experiences with prejudice or discrimination.

Ogbu’s cultural ecological theory has been subject to significant critique, particularly regarding its categorization of minority groups and potential for reinforcing stereotypes (Trueba, 1988). Critics argue that the voluntary/involuntary minority distinction oversimplifies complex immigration experiences and may perpetuate deficit perspectives about certain cultural groups. Additionally, the theory has been critiqued for insufficient attention to within-group diversity and changing generational experiences (Gibson & Ogbu, 1991). Despite these limitations, the theory remains relevant for understanding how cultural background and immigration status shape educational expectations and experiences, particularly for families navigating the model minority myth.

#### 3.1.4. Disharmony Hypothesis

The disharmony hypothesis guides the investigation of the potential for negative teacher attitudes, inadequate support, and the challenges associated with multi-exceptionality to contribute to internal conflicts and maladaptive behaviors in Kent. Interview questions were designed to understand Kent’s socioemotional well-being and any challenges he may face in adjusting to his environment.

The disharmony hypothesis represents one of several competing perspectives on the socioemotional development of gifted individuals. Critics argue that it may pathologize giftedness and perpetuate harmful stereotypes about the “mad genius” (Peterson, 2009). Additionally, empirical support for the hypothesis remains mixed, with some studies finding no significant differences in adjustment between gifted and typical populations (Neihart, 1999). However, for multi-exceptional students facing both the challenges of disability and the complexities of cultural identity, the framework provides a useful lens for understanding how environmental mismatches and inadequate support can contribute to socioemotional difficulties.

#### 3.1.5. Critical Integration of Theoretical Frameworks

The integration of these four theoretical perspectives is not without tensions and potential contradictions. For instance, while the DMGT emphasizes individual talent development, cultural ecological theory highlights how group membership and historical experiences shape educational outcomes, potentially creating tension between individual and collective perspectives. Similarly, Bronfenbrenner’s emphasis on environmental influence may appear to conflict with the disharmony hypothesis’s focus on internal psychological processes.

However, these tensions can be viewed as productive rather than problematic. The multi-dimensional nature of multi-exceptionality requires theoretical approaches that can capture complexity and contradiction. By acknowledging these tensions rather than resolving them, we can better understand the lived experiences of students who navigate multiple, sometimes conflicting identity positions and environmental demands.

Furthermore, each framework contributes unique insights while having distinct blind spots. Bronfenbrenner’s theory excels at mapping environmental influences but may underemphasize individual agency. The DMGT provides a nuanced view of ability development but may not fully account for cultural variations in how abilities are recognized and valued. Cultural ecological theory illuminates group-level experiences but may not capture individual variations within cultural groups. The disharmony hypothesis highlights potential challenges but may not adequately address resilience and positive adaptation.

By employing multiple theoretical lenses while acknowledging their limitations, we aim to provide a more complete, if necessarily complex, understanding of Kent’s experiences. This multi-framework approach proved essential for understanding Kent’s case, as it allowed us to simultaneously examine how his family’s cultural values (cultural ecological theory) created both support and pressure, how his school environment (ecological systems theory) failed to address his complex needs across multiple system levels, how his giftedness and disabilities interacted through various catalysts (DMGT), and how inadequate support contributed to his behavioral challenges (disharmony hypothesis)—insights that would have been incomplete or missed entirely through any single theoretical lens. More critically, this integration reveals dynamic interactions that no single framework could illuminate. For example, while cultural ecological theory alone might identify family pressure, and the DMGT might separately categorize this as an environmental catalyst, their combination reveals how cultural expectations create paradoxical effects. This simultaneously motivates Kent’s intellectual development while generating perfectionism that triggers the very behavioral challenges described by the disharmony hypothesis. Similarly, Bronfenbrenner’s mesosystem analysis becomes particularly powerful when combined with cultural ecological theory to show how home–school cultural mismatches (viewing Kent’s interests as “inappropriate”) create systemic barriers to his talent development. This synergistic analysis produces insights about multi-exceptionality that are genuinely novel, revealing how cultural identity does not simply add complexity to giftedness and disability; it fundamentally transforms how these characteristics manifest and develop.

### 3.2. Data Collection

This single-case study adhered to the rigorous methodological standards outlined by Yin (2014) for conducting qualitative case study research. Data were collected from various sources to enable robust triangulation and provide a comprehensive, holistic understanding of the case. These sources included diagnostic materials, school records spanning one academic year, and semi-structured interviews with key stakeholders.

Diagnostic reports, psychoeducational evaluations, and health records supplied by the family were analyzed to discern Kent’s (the participant’s) abilities, disabilities, strengths, weaknesses, and intervention history. Academic transcripts, individualized education plans (IEPs), progress reports, and teacher notes offered insights into his educational experiences, performance, support provisions, and behaviors.

In-depth interviews were conducted using semi-structured protocols developed specifically for this study (see Appendix A) with a range of individuals to capture diverse perspectives. Kent participated in two recorded interviews lasting 45–60 min, while each of his parents participated in one recorded interview lasting 60–90 min (with an additional follow-up conversation with Kent’s mother). We also interviewed a board-certified behavior analyst and three school staff members (Kent’s case manager, special education teacher, and behavior specialist); each of these interviews lasted around 60 min. All interviews were audio-recorded and professionally transcribed. These interviews allowed us to explore various perspectives and experiences associated with Kent’s talent development, challenges related to disability, cultural identity, and overall educational journey.

Throughout the study, we maintained regular communication via email correspondence with Kent and his family over a period of 12 months. This extended engagement, paired with data source triangulation, facilitated a rigorous, ethical exploration of this complex situation to support the credibility and trustworthiness of our findings.

While comparative cases (such as a same-age child with disabilities but different cultural background, or a culturally similar child without disabilities) could provide additional analytical depth, the single-case approach was selected because Kent’s case represents what Yin (2014) terms a “critical case” that provides significant insights into theoretical propositions about multi-exceptionality at cultural intersections. The complexity of variables (profound giftedness, multiple disabilities, specific cultural background, family immigration history) makes finding truly comparable cases extremely challenging, and our primary purpose is theoretical development rather than comparative analysis.

### 3.3. Data Analysis

Consistent with the exploratory aims of this study, we employed a flexible, qualitative approach to data analysis. This methodological choice was made to enable a nuanced and preliminary understanding of the intersections among giftedness, disability, and cultural identity as they manifested in a single, complex case. Exploratory methods are particularly appropriate in contexts where phenomena are multifaceted and not yet well defined in the literature, allowing for emergent patterns and themes to be identified without imposing restrictive analytic frameworks.

Given the focused, case-based nature of our data—consisting primarily of school records, interviews, and correspondence—the application of systematic content analysis, quantitative coding, or formal statistical summaries was deemed inappropriate for this initial phase. Such methods are typically reserved for later-stage or confirmatory studies with larger and more homogeneous data sets. Instead, we selected approaches that would best illuminate contextual details relevant to this unique case, prioritizing depth of insight over breadth or generalizability.

A systematic, collaborative analytic approach aligned with case study best practices was employed (Yin, 2014). We began by organizing and coding our data, categorizing information based on themes and patterns that emerged from the collected data. This process allowed us to identify commonalities and differences in experiences, strengths, and challenges across the data sources. We then analyzed the data using qualitative analysis techniques to comprehensively understand the phenomenon under study. Data collection and analysis were iterative and responsive; as new dimensions emerged, additional targeted data were gathered to achieve a comprehensive, contextualized characterization. 

Each of the theoretical frameworks also helped shape the data analysis. The ecological systems theory facilitated examinations of interactions between Kent and his family, teachers, and peers (microsystem), the collaboration between home and school (mesosystem), the impact of school policies (exosystem), and the influence of cultural values and beliefs about giftedness and disability (macrosystem). Identifying Kent’s natural abilities (giftedness), how these abilities are nurtured or hindered by intrapersonal factors (e.g., motivation, self-regulation), and environmental factors (e.g., educational opportunities, support systems) to develop talents through the differentiated model of giftedness and talent was also beneficial. The data was coded to identify catalysts and developmental processes that either facilitated or impeded Kent’s talent development.

Analyzing how Kent’s experiences as an Asian American student shaped his educational journey and talent development through the cultural ecological theory contributed to the exploration of potential conflicts between cultural expectations and individual needs, and how these conflicts impact his socioemotional well-being. Finally, the disharmony hypothesis was used to examine negative teacher attitudes, inadequate support, and the challenges associated with multi-exceptionality, which may contribute to internal conflicts and maladaptive behaviors in Kent. Each of these frameworks underscores and structures the importance of individualized socioemotional support to address his unique challenges and promote positive outcomes.

### 3.4. Positionality Statement

Given the qualitative nature of this study, it is essential to disclose our positionality to provide context for our approach and findings. The primary research team consisted of two experienced mixed-methods researchers with clinical backgrounds specializing in twice-exceptionality and multi-exceptionality. Both researchers have disabilities, which informs their understanding of and approach to the subject matter. One researcher identifies as a white immigrant woman from Northwestern Europe, while the other identifies as a Native American man. Their personal experiences with disability and diverse cultural backgrounds contribute to a nuanced understanding of the intersectionality of race, culture, and disability.

Two graduate research assistants supported our research team in writing this paper. One assistant identifies as both white and Native American and is a mother to a twice-exceptional child. This dual perspective as a researcher and a parent of a child with experiences similar to those of our case study subject provides valuable insights. The other assistant is an African American woman from the Southern United States. Both graduate assistants have backgrounds as teachers, which adds an educational perspective to our analysis.

We recognize that our diverse team brings a range of perspectives that can enrich our understanding of the complex interplay between culture, disability, and education. However, we also acknowledge that our collective experiences and identities may not fully align with those of our Asian American participants. We have strived to approach this case study with cultural humility, recognizing the limitations of our own experiences and actively seeking to understand the unique perspectives of our participants through reflexive practices to examine our own biases and assumptions. We also prioritized the voices and experiences of our participants, ensuring that our interpretations were grounded in their lived experiences rather than our preconceptions.

We present this positionality statement to provide transparency about the lens through which we approached this research, allowing readers to engage with our findings and interpretations critically.

## 4. Findings

### 4.1. Kent’s Case Profile

Kent is a nine-year-old Asian American boy who presents with a multifaceted and complex neurodevelopmental profile, characterized by profound giftedness alongside disability. Diagnosed with ASD at the age of three, Kent also displays symptoms that are suggestive of comorbid ADHD and DCD. However, the latter conditions are undergoing assessment.

Kent is the eldest child in a Chinese American family residing in the United States, with two younger twin siblings with developmental delays. His early environment included bilingual exposure to both English and Mandarin. Intellectual assessment conducted using the Wechsler Intelligence Scale for Children–Fifth Edition (WISC-V) reveals an exceptionally high IQ of 144, reflecting precocious cognitive strengths and advanced academic abilities, despite social-communicative challenges associated with ASD that impact aspects of his daily functioning. Kent demonstrates significant fine motor delays and restricted, repetitive behaviors that pose obstacles in various contexts, including home, school, and community. Recently, his maladaptive behaviors have intensified, culminating in specialized classroom placement due to severe verbal aggression, volatility, and threats toward peers. These behaviors appear to stem partly from the frustration of navigating the polarity between his high intellectual capacity and areas of disability, posing unique challenges for Kent and his devoted family.

An array of intensive interventions has been designed to target Kent’s diverse needs since infancy, including applied behavior analysis (ABA), occupational therapy, speech-language pathology, social skills training, counseling, and special education classroom support. Kent’s parents have been staunch advocates in securing services through both public and private avenues to meet their son’s needs. These interventions aim to address Kent’s complex profile, supporting his gifted abilities while targeting areas of challenge.

Kent’s comprehensive intervention history also included consideration of pharmaceutical options. The school team recommended consultation with medical professionals regarding ADHD medication, recognizing the potential benefits for attention and behavioral regulation. However, Kent’s family made a deliberate decision to delay pharmaceutical interventions, with his mother stating her preference to “hold off on medication for two more years” due to concerns about medicating young children. This decision reflects the complex treatment considerations that multi-exceptional families navigate, balancing potential benefits of medication against developmental, cultural, and personal factors. The family’s approach prioritized intensive behavioral interventions and counseling support while maintaining the option to revisit pharmaceutical treatments as Kent matured.

### 4.2. Kent’s Strengths

As evidenced by his profile, Kent exhibits numerous strengths, including intellectual curiosity, creativity, and a capacity for empathy when assuming teaching roles. Kent’s school social worker/case manager (denoted as “MM”), special education teacher (denoted as “MS”), and behaviorist (denoted as “JS”), each recounted that “…He has a lot of theories about things and how to like, make things better”, and that “…when he feels like he can model appropriate behavior and show someone something or teach someone something like he wants to put on presentations for the class…it’s very kind and he is very sweet with them”. These behaviors not only demonstrate Kent’s high cognitive ability but also may indicate his ability to communicate effectively with his peers in educational settings, as Kent’s behavior and intellect flourish when he is placed in a leadership role where he can share his knowledge. MM observed that Kent has demonstrated strengths with other adults, particularly in his insightful conversations and above-grade-level abilities in mathematics. This capacity to engage in meaningful dialogue with peers and adults further highlights Kent’s cognitive giftedness and potential for advanced academic achievement. 

Nevertheless, Kent not only exhibits these strengths in educational environments but also within the home. When discussing Kent’s strengths with his parents, they highlighted qualities like perseverance, self-motivation, and memory. His ability to persist through challenges, coupled with his intrinsic drive to learn, reflects characteristics often associated with high-ability learners. These parental insights into Kent’s attributes not only offer a more holistic understanding of his abilities in different contexts but also highlight his resilience and academic success despite the challenges associated with his neurodevelopmental profile.

#### Considerations Contextualizing Challenging Behaviors

While Kent’s challenging behaviors primarily manifested as responses to frustration and unmet needs, some patterns warrant careful consideration within the broader literature on behavioral development in gifted populations. Kent’s occasional use of threatening language, interest in violent topics, and apparent ability to manipulate situations for desired outcomes bear some superficial resemblance to traits associated with the “dark triad” of personality characteristics (narcissism, machiavellianism, and psychopathy) that have been explored in gifted populations (Kowalski et al., 2018; Matta et al., 2019).

However, several critical factors must be emphasized. First, Kent’s young age makes any personality-based interpretations highly inappropriate and potentially harmful. Second, his behaviors appeared contextually driven rather than pervasive, emerging primarily during periods of stress, frustration, or perceived failure. Third, Kent demonstrated genuine empathy, remorse, and desire for positive relationships, which contrasts sharply with true dark triad presentations. Most importantly, when his educational and emotional needs were better met, these concerning behaviors diminished significantly.

Rather than representing inherent personality traits, Kent’s challenging behaviors likely reflected adaptive responses to an environment that inadequately addressed his complex needs. His threats and manipulation may have represented his most effective available strategies for communicating distress and regaining control in overwhelming situations. This interpretation aligns with trauma-informed and neurodevelopmental perspectives that view challenging behaviors as symptoms of unmet needs rather than character flaws.

This case highlights the critical importance of avoiding pathologizing interpretations of challenging behaviors in multi-exceptional children, instead focusing on comprehensive assessment and intervention that addresses underlying needs while building more adaptive coping strategies.

### 4.3. Individual Needs

Kent’s mother revealed that Kent had been receiving intensive applied behavior analysis (ABA) since he was two years old and currently receives support from school and at home from ABA specialists. However, even this specialized training does not always appropriately meet his needs. Kent’s mother expressed significant concerns regarding the intense focus on Kent’s disability, which often overlooks his intellectual abilities and strengths, noting:

…at the end of [the] day, he’s still saying the same…That’s my concern is that they know he’s smart, but they forget just because they are very good ABA people. … they are great ABA people, but they are not. They are not experienced [in] twice exceptional…

This simultaneous inattention to Kent’s strengths and hyperfocus on his disability illustrates the unique challenges that multi-exceptional students like Kent face: a lack of truly individualized support. Kent’s mother further expressed this sentiment when saying, “…last year when because of [Kent]’s… tantrums, the school almost wanting to even pull him out of this trip placement” (1C, p. 9). This recurring theme of inability to meet Kent’s individual needs demonstrates a critical gap in appropriate programming that addresses both Kent’s disability and giftedness, highlighting a prominent issue in the programming available for multi-exceptional students, as well as gaps in the training of professionals who provide this programming. Unfortunately, these issues are exacerbated due to the absence of gifted education programming in Kent’s schools. As his mother noted in the interviews, the ABA that Kent receives is not enough. There is an expressed need for more individualized programming and counseling; this pattern of inadequacy in Kent’s programming may be a contributing factor to his challenging behaviors. 

### 4.4. Challenging Behaviors

While Kent’s strengths are evident in this case study, his challenging behaviors were equally notable. These behaviors, ranging from disruptive classroom conduct to concerning interests, present significant obstacles to Kent’s educational and social development. As noted by several individuals, these behavioral issues can become severe if not addressed appropriately. The district behaviorist who worked with Kent recalls:

…but we saw challenging behavior from…situations not going the way that he perceived that they should, from getting questions wrong…not being [the] first person in line and people working too slowly…and now he’s accessing it [general education] virtually from the ABA classroom due to some more recent challenging behaviors…

Coupled with information about his lack of appropriate programming, Kent’s challenging behavior may represent manifestations of unmet needs. According to the board-certified behavior analyst (BCBA) assigned to oversee Kent’s services, Kent’s behavior often presents as tantrums, property destruction, aggression, and noncompliance, but they have not occurred in sessions frequently. Nevertheless, when they occur, these behaviors cause challenges in the classroom and with Kent’s peers. This reveals a significant gap in Kent’s educational experiences and talent development. While ABA interventions may help manage his behavioral challenges, they do not actively nurture his strengths and talents. Conversely, the general education classroom may support his cognitive abilities but falls short in meeting his behavioral needs. 

Kent’s school team and BCBA specifically mention challenges with verbal aggression towards peers and adults, as well as a heightened interest in violence. Although they voiced frustration with ongoing risk assessments, as Kent typically does not display physical aggression, they clearly expressed their belief that an alternative setting was needed to address his inappropriate comments and better support his needs. MM, his school social worker, expounded upon this by saying,

So, you know, when [Kent] is in there, there’s a lot of…inappropriate comments that could cause…risk. So… he will say to the teacher, ‘I’m going to kill you”, but…he’ll blurt it out to the whole classroom. So, everyone hears that, you know, threading [threatening] things…like, “I’m gonna poison you.

Though alarming, this behavior may once again reflect Kent’s unmet needs within the educational environment. Furthermore, a lack of understanding of how to address his behavior is evident, often leading to his removal from the classroom, making successful reintegration even more difficult. This difficulty is further highlighted by Kent’s special education teacher, who observed that his interests frequently drift into troubling territory:

…[his interests] tend to be on the inappropriate side of the topic…It’s just like ‘oh, I want to know about the worst thing about that country, and then share that information with the world’ that’s why it is kind of difficult…

While we recognize the complexities of managing Kent’s behaviors in the classroom, it is also important to consider the tension surrounding culturally defined notions of “inappropriate” topics and whether Kent fully grasps these nuances within the educational environment. If he does not, these potential conflicts could be contributing to his social and emotional challenges in the classroom. Considering this, when we inquired with Kent’s BCBA about the possible root causes of this behavior, they indicated that Kent may be seeking the attention that these behaviors elicit. Kent’s district behaviorist appeared to align with this perspective, stating that Kent desired “…all that type of negative attention. And I know mom has expressed that she does feel like he likes sometimes that negative attention…” However, they concluded that they have not observed this behavior sufficiently to make a confident claim. Regardless, attributing Kent’s behavior to a desire for attention may, once again, stem from a hyper-focused view of his disability, rather than exploring how to appropriately address his needs. 

#### 4.4.1. Self-Awareness of Challenging Behavior

Kent’s school team has noted his ability to reflect on his behavior retrospectively, which appears to advance his progress positively despite his challenges. His special education teacher has observed that “…he is making more friends in the general education classroom and likes initiating social interactions in a kind and age-appropriate way to play with other students. And I think that’s becoming like he’s getting stronger in that area…” This finding is generally positive, suggesting that Kent has developed an understanding of how his interactions and behaviors affect both the environment and other participants within it. Conversely, the district behaviorist asserts that Kent’s reflections sometimes appear “automatic”, leading them to question whether these reflections are genuine or potentially manipulative. Reflecting a weak understanding of giftedness in this statement, gifted students may cognitively process their actions and their consequences more quickly than their peers, highlighting the gap in training for education professionals working with multi-exceptional students.

The professionals in Kent’s team are not the only individuals who note this challenging behavior; Kent himself demonstrates some knowledge regarding his behavioral challenges. In an interview, he mentioned, “The disruptive behavior that I keep doing…Yeah, my disruptive behavior is turning my classroom into a zoo”, indicating a degree of self-awareness in Kent regarding not only his own behavior, but also how his behavior affects the classroom environment. When the interviewers asked Kent to elaborate, he specifically mentioned his tantrums and the recurring teasing of other students’ ideas. Though these behaviors negatively affect Kent’s development of peer relationships, they may, once again, be byproducts of inadequate support in his educational environment and Kent’s response to the inadequacy of this support. 

#### 4.4.2. Considerations Concerning Behavioral Patterns

While Kent’s challenging behaviors primarily manifested as responses to frustration and unmet needs, some patterns warrant careful consideration within the broader literature on behavioral development in gifted populations. Kent’s occasional use of threatening language, interest in violent topics, and apparent ability to manipulate situations for desired outcomes bear some superficial resemblance to traits associated with the “dark triad” of personality characteristics (narcissism, machiavellianism, and psychopathy) that have been explored in gifted populations (Kowalski et al., 2018; Matta et al., 2019).

However, several critical factors must be emphasized. First, Kent’s young age makes any personality-based interpretations highly inappropriate and potentially harmful. Second, his behaviors appeared contextually driven rather than pervasive, emerging primarily during periods of stress, frustration, or perceived failure. Third, Kent demonstrated genuine empathy, remorse, and desire for positive relationships, which contrasts sharply with true dark triad presentations. Most importantly, when his educational and emotional needs were better met, these concerning behaviors diminished significantly.

Rather than representing inherent personality traits, Kent’s challenging behaviors likely reflected adaptive responses to an environment that inadequately addressed his complex needs. His threats and manipulation may have represented his most effective available strategies for communicating distress and regaining control in overwhelming situations. This interpretation aligns with trauma-informed and neurodevelopmental perspectives that view challenging behaviors as symptoms of unmet needs rather than character flaws.

This case highlights the critical importance of avoiding pathologizing interpretations of challenging behaviors in multi-exceptional children, instead focusing on comprehensive assessment and intervention that addresses underlying needs while building more adaptive coping strategies.

### 4.5. Home Life and Values

#### 4.5.1. Cultural Values

Outside of the classroom, Kent’s school team, BCBA, and even his parents note a complex interplay among family background, cultural identity, and his behavioral challenges. Kent’s BCBA recalled that “…they [Kent’s parents] are so vigilant about trying to integrate, like moral lessons and like, manners and things like that…” Cultural influences, such as honor and respect, significantly shape family values. Therefore, it is not unreasonable to assume that Kent’s family might encourage these behaviors, as evidenced by his mother frequently prompting him to say, ‘thank you’ and ask the interviewers questions during his interview.

Nevertheless, Kent’s family often expresses a sense of dissonance between their traditional family culture and the culture they have adopted since immigrating. Kent’s father expressed that “…when I came to America… people were just so freeform. I mean… just the kids don’t see the same thing… when going to Sunday school, Chinese school, and the other kids were Chinese. They were just like Americans…” This tension experienced by Kent’s parents may reflect similar pressures that Kent himself faces in retaining his family’s traditional culture, particularly in situations where aspects of that culture are considered ‘inappropriate’ in educational settings. Demonstrated when Kent was asked about his reading preferences, he mentioned that “I like to read our big stat books that think America is a bad country. Books that like China’s ideas. Books that respect the Chinese Communist Party…” However, because Kent’s family has immigrated into a culture that may not align with his cultural ideals—evident in his school team’s discussion of his challenging behavior and “inappropriate” topics—he may experience direct conflict with his educational environment, ultimately stifling his growth and strengths.

Kent himself has observed some of these direct conflicts. Elaborating in his second interview, Kent pointed out that he often perceived a divergence between his parents’ ideals and his own and disagreed with the idea of aligning with American ideals rather than maintaining Chinese ideals. Furthermore, these ideals are not only present at home, but also at school, where Kent revealed that he often teases peers because of what they think, specifically concerning positive views about America and American culture, and confesses that he often feels that his family should have remained in their home country. These ongoing tensions between Kent’s home culture and those prevalent in American society present a significant source of stress for Kent both at home and at school, potentially exacerbating his behavioral challenges and complicating efforts to meet his individualized needs.

#### 4.5.2. Academic Values

Academic achievement emerges as another area where familial values and expectations play a significant role. When asked about the importance of doing well in school, Kent responded, “…doing well in school is the only way I can achieve… my mom’s biggest dream… to go to Harvard…” Although Kent’s mother consistently denied this claim, the statement underscores a possible underlying pressure Kent may be facing. This interpretation is supported by observations from Kent’s BCBA, who indicated that while Kent’s parents are keen on nurturing his many talents, it would be beneficial to:

…not sort of put so much pressure on him in these different areas. Like, I wouldn’t say she puts pressure on him in saying ‘you have to do the best where you need to’…It [is] just that putting him in so many things work against his nature…it is a lot of pressure…

Though the BCBA acknowledges this pressure and encourages Kent’s mother to ease her expectations, it is evident that both the home culture and adopted culture, and the conflict therein, place significant pressure on Kent. His district behaviorist offered more context for why this might be, suggesting that perfectionism and being the oldest in his family may apply pressure on him as well, saying,

…And even mom will say, you don’t have to do that. And he’s like, “No, I have to do it. I have to do it” and it’s like…he puts so much pressure on himself…But when he’s not the best at it, he has a hard time tolerating that…

This indicates that Kent may recognize the real or perceived familial standards of cultural tradition and gender, as discussed in prior literature. This recognition may prove difficult for him to align with his own performance, potentially triggering challenging, maladaptive behaviors. These identified pressures—cultural dissonance, academic expectations, and perfectionism—could explain how external expectations and internal conflicts may contribute to the intensity of Kent’s behavioral challenges. 

#### 4.5.3. Emotional Impact

A missing key element in many of the earlier discussions is the complex emotional “tax” felt by families who support multi-exceptional students. When interviewing Kent’s BCBA and his case manager, they mentioned Kent’s mother’s frustration several times, particularly regarding his use of threatening language, despite having limited access to media. This frustration highlights not only parents’ challenges in managing and understanding their multi-exceptional child’s behaviors—especially when they seem incongruent with the child’s environment or upbringing—but also a lack of support available to help them navigate these challenges. Kent’s father also hints at the fears he and Kent’s mother experienced when first receiving Kent’s diagnosis: “So, she was like really scared and had gotten scared…And yeah, in the beginning, I remember getting quite scared. Now I still kind of have my doubts about if [Kent] can be fully independent…” This candid reflection reveals the ongoing anxiety and uncertainty parents of multi-exceptional children may experience regarding their child’s development and future. Reconciling Kent’s imagined life goals with the realities of his educational needs serves as a constant reminder to his family that their aspirations for him may be unrealistic or, at best, exceptionally difficult to achieve. This tension between hope and reality often fuels strong emotional responses from both Kent and his parents.

A particularly touching moment emerged during the interview with Kent’s father, where he discussed Kent’s experience with a principal. He explains: “…the gist of it was, he [the principal] was trying to tell them that… you have to do well in school…. and if you don’t, then you’ll be homeless…” Kent’s father later elaborated on how Kent reacted to this interaction, in which Kent’s father shed tears. This emotional response suggests the deep impact educational experiences and interactions can have on multi-exceptional children and their families, further highlighting the need for sensitive, individualized, and culturally informed approaches from educational professionals.

Although these accounts reflect themes of frustration, uncertainty, and emotional vulnerability—highlighting the emotional toll of supporting multi-exceptional students—they also underscore the need for appropriate interventions that support both these students and their families. These experiences emphasize the importance of addressing multi-exceptional children’s needs while also providing culturally responsive emotional support and resources for the entire family.

## 5. Discussion

Kent’s case provides a rich empirical foundation for examining how giftedness, disability, and cultural identity intersect within complex environmental systems. The integration of our four theoretical frameworks reveals insights that extend beyond what any single lens could illuminate, demonstrating both the value and limitations of existing theoretical approaches to multi-exceptionality.

### 5.1. Cultural Microsystem Conflicts and Environmental Mismatch

Bronfenbrenner’s ecological framework proves particularly revelatory in understanding how Kent’s challenging behaviors emerge from systematic environmental mismatches across multiple system levels. At the microsystem level, Kent experiences conflicting cultural messages between home and school environments. His interest in Chinese political ideology and criticism of American values, encouraged within his family’s cultural framework, becomes labeled as “inappropriate” within the school microsystem, creating chronic stress and behavioral responses.

The mesosystem analysis reveals significant home–school disconnections that extend beyond typical parent–teacher communication issues. Kent’s family values academic excellence and cultural preservation, while his school environment prioritizes behavioral compliance and American cultural norms. This mesosystem conflict manifests in Kent’s confusion about appropriate behavior and contributes to his escalating challenging behaviors when he cannot reconcile these competing expectations.

At the exosystem level, school policies that lack cultural responsiveness indirectly impact Kent’s daily experiences. The absence of gifted programming, combined with ABA interventions that focus solely on deficit-based behavioral modification, reflects broader educational policies that fail to accommodate multi-exceptional, culturally diverse students. These policy gaps create systemic barriers to appropriate support.

The macrosystem influence is evident in how broader American cultural values about appropriate childhood interests and behaviors conflict with Kent’s family’s cultural framework. The “model minority” stereotype creates additional pressure, as educators expect academic compliance without recognizing the cultural complexity of Kent’s experiences or the interaction between his giftedness and disabilities.

This analysis extends Bronfenbrenner’s framework by revealing what we term “cultural microsystem displacement”—where immigrant families’ home microsystems exist in constant tension with institutional macrosystems, creating unique developmental challenges not adequately captured in traditional ecological applications. Kent’s case suggests that for culturally diverse students, the microsystem itself may be culturally bifurcated, requiring a more nuanced understanding of environmental influences.

### 5.2. Cultural Catalysts and Paradoxical Development Processes

Gagné’s DMGT framework highlights how Kent’s giftedness and talent development are influenced by complex catalyst interactions; however, his case also reveals limitations in the model’s conceptualization of cultural factors. Kent demonstrates clear natural abilities (giftedness) in intellectual domains, evidenced by his IQ of 144 and advanced reasoning capabilities. However, the transformation of these abilities into developed competencies (talents) is significantly complicated by the interaction of cultural catalysts with his disabilities.

Environmental catalysts in Kent’s case function paradoxically. His parents’ high academic expectations and cultural emphasis on educational achievement serve as positive catalysts for intellectual development, motivating his advanced academic performance. Simultaneously, these same expectations create pressure that exacerbates his ADHD symptoms and triggers behavioral challenges, effectively functioning as negative catalysts for socioemotional development.

Intrapersonal catalysts reveal similar complexity. Kent’s motivation and perfectionism, likely influenced by both his giftedness and cultural values, drive academic achievement while creating anxiety and behavioral rigidity when he cannot meet impossibly high standards. His self-awareness of his behaviors demonstrates metacognitive strength; yet, this same awareness may contribute to his distress about not meeting expectations.

The developmental process is further complicated by what we term “catalyst interference”—where positive catalysts for giftedness development simultaneously impede disability-related needs. For example, accelerated academic placement capitalizes on his intellectual abilities but provides inadequate support for his social communication challenges and sensory needs associated with ASD.

Kent’s case suggests the DMGT may need refinement for culturally diverse populations where traditional catalyst categories blur. Cultural identity functions as both an intrapersonal and environmental catalyst simultaneously, creating complex interactions not clearly delineated in Gagné’s original framework. This finding calls for a more nuanced understanding of how cultural factors operate within talent development models.

### 5.3. Model Minority Paradox and Exceptionality Dissonance

Ogbu’s cultural ecological theory provides crucial insights into how Kent’s family’s voluntary immigrant status shapes their educational approach, while also revealing limitations when applied to multi-exceptional students. Kent’s parents demonstrate the optimistic educational expectations Ogbu associates with voluntary minorities, viewing education as a pathway to success and maintaining a strong cultural identity while adapting to American systems.

However, Kent’s multi-exceptional profile creates what we term “exceptionality dissonance” within the model minority framework. His giftedness aligns with cultural expectations and stereotypes about Asian American academic achievement, while his disabilities challenge family and cultural narratives about success and achievement. This creates internal family conflict not anticipated in Ogbu’s original framework.

The case reveals how disability disrupts cultural ecological predictions about voluntary minorities’ educational success. While Kent’s family maintains optimistic expectations, his ASD and ADHD diagnoses create uncertainty about future independence and achievement, leading to the emotional distress evident in interviews with his parents. The family struggles to integrate their cultural values of academic excellence with the reality of Kent’s complex needs.

Furthermore, Kent’s own cultural identity development is complicated by his disabilities. His preference for Chinese political ideology and criticism of American values may represent both cultural identity formation and a response to feeling alienated from American educational environments that cannot accommodate his needs. This suggests that cultural identity development in multi-exceptional students may follow different trajectories than those predicted by standard applications of cultural ecological theory.

The model minority myth creates additional complications, as educators may attribute Kent’s challenges to behavioral choices rather than recognizing underlying disabilities, consistent with research by Park and Foley-Nicpon (2022). This misattribution delays appropriate support and contributes to the cycle of behavioral escalation observed in Kent’s case.

### 5.4. Connections to Broader Multi-Exceptionality Literature

Kent’s case contributes to growing literature on the complexity of multi-exceptional students while highlighting significant gaps in research addressing cultural intersections. His experiences align with research by Foley-Nicpon et al. (2013) demonstrating the need for individualized approaches that address both strengths and challenges, while extending this work to show how cultural factors complicate traditional twice-exceptional frameworks.

The case supports findings by Gelbar et al. (2022) about the complexity of gifted students with ASD, while revealing additional layers when cultural identity intersects with these characteristics. Kent’s profile demonstrates how cultural values can both support and complicate the development of students with ASD, particularly around social communication expectations and behavioral norms.

Kent’s experiences with inadequate educational programming align with research by Assouline and Whiteman (2011) showing how giftedness and disability are often addressed in isolation. However, his case extends this concern to include cultural responsiveness, suggesting that truly individualized programming must consider the intersection of ability, disability, and cultural identity simultaneously.

The family’s emotional impact and advocacy efforts connect to broader literature on families of multi-exceptional students (Arnstein, 2020, 2023), while highlighting unique challenges faced by immigrant families navigating unfamiliar educational systems while preserving cultural identity. Kent’s parents’ struggles with accepting his disabilities while maintaining high expectations reflect broader tensions documented in research on Asian American families (Park et al., 2018).

These theoretical contributions highlight the need for more sophisticated models that can capture the dynamic interactions between ability, disability, and cultural identity in educational contexts. They also point toward practical implications for developing more effective, culturally responsive approaches to supporting multi-exceptional students and their families.

## 6. Implications and Recommendations

While Kent’s case offers significant insights, it is essential to clarify that this single case study is not intended for broad generalization. Kent’s situation exemplifies an unusual and complex interaction between giftedness, multiple disabilities, and cultural factors. The unique nature of Kent’s behaviors and challenges makes this case a compelling focus for research; however, direct application of these findings to other contexts requires careful consideration. Nevertheless, Kent’s case underscores the potential of strength-based, talent-centered approaches for multi-exceptional students and highlights the critical need for individualized assessment and intervention in such intricate cases (R. Olenchak, 2017).

### 6.1. Relevance of Comprehensive Assessment

First, Kent’s case demonstrates the necessity of conducting comprehensive assessments when working with multi-exceptional students. In the interviews with Kent’s parents, school team, BCBA, and himself, it became increasingly evident that Kent may be grappling with a profound pressure to excel within and beyond the home. While the root causes of this pressure were not explicitly stated, it is apparent that the family’s cultural dynamics and subsequent values play a significant role. This discovery was possible due to the diverse viewpoints offered by Kent, his family, educators, and caseworkers. We assert that addressing the needs of multi-exceptional students is not a solo sport and extends well beyond the endeavors of individual teachers (Kirk et al., 2017). It necessitates a collaborative approach involving education professionals and students’ families, as described in Baum et al.’s (2014) Multiple Perspectives Process Model (MPPM). Because multi-exceptional students’ stories often vary in degrees of complexity, it is critical to comprehensively understand the child’s history and family dynamics—incorporating a range of perspectives—to ensure that parents and relevant caregivers are aware of what to expect and are aligned concerning tailored interventions and support strategies (Coleman & Gallagher, 2015).

### 6.2. Cultural Competence in Multi-Exceptionality 

As discussed in the literature review, the traditional emphasis on male leadership found in East Asian cultures often translates into high expectations for eldest sons, who are nurtured from a young age to carry the family’s legacy and uphold its reputation (Seow, 2020). For multi-exceptional children like Kent, these cultural pressures can create unique challenges as they navigate both their exceptional abilities and their disabilities. In first-generation immigrant families like Kent’s, this pressure may intensify due to the desire to preserve their cultural identity. The family’s aspirations often converge on the success of the eldest son, creating a significant burden, particularly for children with unique learning needs (Sechiyama, 2013). This pressure to achieve can trigger behavioral issues and challenges in both academic and social settings. 

When children struggle to align with traditional cultural ideals, families may experience a delayed and extended grieving process, potentially delaying critical interventions and accommodations (Shao & Lee, 2023). While understandable, this tendency to hold onto cultural ideals can impede timely access to support the child’s individual needs. Although traditional gender expectations are evolving across many East Asian communities, they remain deeply influential, especially within immigrant families preserving cultural connections (Seow, 2020). Kent’s mother’s frequent corrections during interviews exemplify this struggle, illustrating the tensions between cultural values and the acceptance of a child’s unique needs and abilities. 

This complex interplay between cultural background, familial expectations, and individual traits underlines the need for culturally competent assessments and interventions. Kent’s experience highlights the importance of an educational approach that fully acknowledges his culture alongside his multi-exceptional profile. For professionals, cultural competence is crucial in recognizing and respecting the nuances of cultural identity while navigating the intersections of giftedness and disability, ensuring that multi-exceptional students from diverse backgrounds receive comprehensive and effective support. 

### 6.3. Limitations of Applied Behavioral Approaches

Kent’s case demonstrates the multifaceted treatment decisions that families of multi-exceptional children must navigate. Beyond behavioral and educational interventions, the family considered pharmaceutical options for ADHD symptom management. The school team’s recommendation for medical consultation regarding medication represents standard practice for addressing attention and behavioral challenges. However, the family’s decision to delay pharmaceutical interventions reflects legitimate concerns about medicating young children and highlights the importance of family autonomy in treatment decisions.

This treatment consideration adds another layer to the complexity of supporting multi-exceptional students. Families must weigh potential benefits of medication against developmental considerations, cultural values, and personal preferences. In Kent’s case, the family’s preference for intensive behavioral interventions and counseling support, while maintaining future flexibility regarding medication, represents one valid approach among many possible treatment configurations.

Healthcare providers and educators should respect family decision-making processes while ensuring access to comprehensive information about all treatment options, including their benefits, risks, and interactions with other interventions.

Kent’s case emphasizes the potential insufficiency of ABA for profoundly gifted individuals with disabilities. Though not obsolete—as Kent’s school team and BCBA reported some progress in Kent’s behavior—ABA’s lack of intellectual rigor and its medical model approach may not align with the unique needs and strengths of multi-exceptional students. This finding aligns with existing research, which frequently has concluded that giftedness and disability status are addressed in isolation from one another (Assouline & Whiteman, 2011), with the emphasis predominantly on addressing disability concerns (Crim et al., 2008; Assouline & Whiteman, 2011). Despite making significant progress in developing peer relationships and engaging in self-reflection, Kent’s challenging behavior frequently resulted in his isolation from the general education classroom, confining him to virtual access. This experience exemplifies how an overemphasis on disability can substantially hinder students’ potential (Baum et al., 2014; King, 2005). Therefore, we advocate for a collaborative approach among education professionals and other stakeholders, such as counselors and ABA specialists, to effectively explore alternative interventions for multi-exceptional students. 

### 6.4. Strength-Based, Talent-Centered Approaches

Furthermore, we recommend adopting strength-based, talent-centered approaches that lead to better outcomes for multi-exceptional students. As emphasized by various individuals in the case study, Kent is an exceptionally bright student, whose strengths often go unnoticed because of the severity of his behavior and disability. However, prior literature supports recognizing and nurturing an individual’s unique abilities and interests, which can promote their overall well-being and personal development (Baum & Olenchak, 2002; Baum & Owen, 2004; Foley-Nicpon et al., 2011; Reis et al., 2014/2014; F. R. Olenchak, 1995). Weinfeld et al. (2005) asserted that students with gifts and talents, who also have disabilities, gain maximal benefit from their programs when both accommodation for deficits and focus on their strengths are incorporated. Similarly, Baum and Olenchak (2022) concluded that consistently using strength-based, talent-centered approaches supports the positive psychology principle to foster academic and affective development. Furthermore, the consistent recognition across various contexts—school, therapy sessions, and home—illustrates the importance of a holistic approach in supporting twice-exceptional children like Kent. By acknowledging students’ strengths as leverage points, educational strategies can better nurture their gifts while addressing areas of difficulty.

### 6.5. Individualized Approaches

Alongside recommending strength-based, talent-centered approaches, we also desire to advocate for individualized approaches for multi-exceptional students. As observed in the case study, addressing challenging behavior poses significant hurdles, particularly in school settings. Prior literature highlights teachers’ attitudes towards inclusive classrooms, where they may lack the support, resources, and training required to serve students with multiple exceptionalities (Parey, 2019, 2021). Moreover, the challenging behaviors and the lack of investigation in this case study hindered Kent’s ability to safely engage with his peers socially, a common finding in earlier research (Avramidis et al., 2018; Schwab, 2015).

Kent demonstrated curiosity across subjects, yet his attention often became fixated on disturbing topics, which raised concerns among his peers and teachers. For example, rather than focusing on cultural achievements or historical contexts of Chinese history, he developed an interest in authoritarian figures, torture methods, and their applications across contexts. While his teacher noted this unusual interest, neither educators nor school counselors explored the possible motivations behind Kent’s focus on these darker elements.

Unfortunately, because these behaviors were not further investigated, they left a significant question unanswered: was Kent’s interest in dark topics indicative of an internal emotional struggle or a means of seeking attention within a system he found alienating? His behavior escalated when he began making threatening remarks to classmates about hurting them—an alarming development that also went uninvestigated. As a gifted preadolescent, Kent shows self-awareness regarding how his actions impact the classroom environment. It is possible that he learned such remarks could potentially lead to his removal from class, providing an escape from situations he found uncomfortable or overstimulating. Without guidance to understand these behaviors, Kent’s underlying needs remained unaddressed.

Though the level of violence observed presents notable challenges for serving the child in traditional school settings, we emphasize that these behaviors need to be further contextualized with Kent’s age in mind. A fully individualized approach, such as a specialized day institution with appropriately trained teachers and staff, may be essential to ensure the child’s safety and provide adequate support. (Assouline et al., 2006).

### 6.6. Holistic Family Support

We recommend implementing comprehensive family support systems for children with multiple exceptionalities. Kent’s case revealed a significant emotional impact on his family, characterized by frustration, uncertainty, and anxiety about his future. Research suggests that many Asian and Asian American families may struggle with feelings of deficit labeling, guilt, and self-blame regarding their children’s disabilities (Choi & Ostendorf, 2015; Yan et al., 2017). This dynamic may indicate a reluctance to fully accept these disabilities, which can hinder their ability to seek necessary support and resources. Interviews revealed that Kent’s parents held aspirations for him that aligned with traditional cultural values; however, it remains unclear whether they recognize and have accepted that these expectations may inadvertently create pressure, leading to challenging emotional responses from Kent. 

Therefore, we assert that the interplay of cultural expectations and values requires that cultural competence be embedded within parental support initiatives. This approach is essential to effectively meet the diverse needs of families through bidirectional systems of support and communication for multi-exceptional students (Arnstein, 2020, 2023; Davis, 2014). These supports should include counseling, education, and resources for parents and siblings to help them navigate the unique challenges of raising a multi-exceptional child.

### 6.7. Long-Term Planning and Support

Given Kent’s parents’ concerns about his future independence, it is critical to emphasize the importance of long-term planning for multi-exceptional individuals. Currently, Kent has transitioned to a specialized school that is better equipped to address his unique needs. Nevertheless, we emphasize prior literature regarding the benefits of strength-based approaches to talent development and disability support (Foley-Nicpon et al., 2013; Gierczyk & Hornby, 2021; Jacobs, 2020). Thus, we contend that specialized schooling should not represent the conclusion of Kent’s educational journey. With specialized interventions over time, we hypothesize that Kent may potentially reintegrate into the general education classroom. Recommendations for this outcome include developing transition programs and ongoing support systems as these students move into adulthood, ensuring they have the necessary skills and resources for independent living and career success.

## 7. Limitation

This exploratory study focused primarily on the most salient factors affecting Kent’s case through available school records, interviews, and correspondence. While this approach enabled deep qualitative analysis, it inherently limited the breadth of our investigation. We were unable to conduct a comprehensive examination of environmental and peer group influences, which are recognized as critical factors in the development and expression of giftedness and disability. These dynamics were addressed only indirectly, constraining our understanding of their full impact.

The single-case design limits generalizability, as individual experiences vary widely across different cultural, educational, and familial contexts. Our qualitative, exploratory methodology prioritized contextual depth over statistical rigor, forgoing systematic content analysis and quantitative measures that might enhance future studies. Additionally, the scope and nature of available data sources may have restricted the range of perspectives captured regarding Kent’s development and support systems.

These findings should be interpreted as preliminary insights intended to inform future research rather than definitive conclusions. Subsequent studies would benefit from broader environmental analysis, mixed-methods approaches, and larger, more diverse samples to better understand the complex interplay among giftedness, disability, and cultural identity.

## 8. Conclusions

Kent’s case study shows the complex interplay among giftedness, multiple disabilities, and cultural factors in shaping the experiences of multi-exceptional individuals. While the extreme nature of this case limits its generalizability, it serves as a catalyst for critical reflection on current practices and future directions in supporting multi-exceptional learners.

This study highlights the urgent need for a paradigm shift in how we approach individuals with multiple exceptionalities. Moving beyond siloed interventions that focus solely on deficits or talents, we must embrace holistic, culturally responsive strategies that honor the full spectrum of an individual’s abilities and challenges. The development of such approaches requires interdisciplinary collaboration among educators, mental health professionals, and researchers to create comprehensive, personalized support systems that accentuate strengths while acknowledging disabilities, but de-emphasize them.

Furthermore, this case highlights significant gaps in our understanding of multi-exceptionality, particularly in relation to extreme behaviors and potential resemblance to manipulation and, in extreme cases, dark triad traits. Future research should delve deeper into these areas, exploring how such behaviors may potentially manifest and be used when students’ needs are not being effectively met within educational settings. Future research should also develop work towards professional development and training for teachers and other specialists to address root causes of these behaviors, and not simply the behaviors themselves, to promote positive development and student advocacy. 

Ultimately, the goal is to foster environments where individuals like Kent, who are multi-exceptional, can thrive, leveraging their unique strengths while addressing their challenges. This necessitates ongoing advocacy for policy changes, increased resources, and enhanced professional development to better equip educators and caregivers.

## Data Availability

The data presented in this study are not publicly available due to privacy and confidentiality concerns related to participant information. Requests for access to anonymized or aggregated data may be directed to the corresponding author and will be considered on a case-by-case basis in accordance with institutional ethics requirements and applicable data protection regulations.

## Appendix A. Semi-Structured Interview Protocols

(All items are intended to serve only as guides for deeper conversations, the direction of each being steered by those being interviewed.)

Parent(s)

1. Describe Kent’s strengths.
2. Describe Kent’s challenges.
3. Tell me the family’s greatest strengths where Kent is concerned.
4. Tell me the family’s greatest challenges where Kent is concerned.
5. Describe a perfect day where Kent is concerned.
6. Describe a bad day where Kent is concerned.
7. Tell me three things you wish for where Kent is concerned.
